# Long Lasting Antibodies From Convalescent Pertussis Patients Induce ROS Production and Bacterial Killing by Human Neutrophils

**DOI:** 10.3389/fcimb.2022.888412

**Published:** 2022-05-12

**Authors:** Michiel M. Kroes, Lars C. van Vliet, Ronald H. J. Jacobi, Betsy Kuipers, Daan K. J. Pieren, Alberto Miranda-Bedate, Cécile A. C. M. van Els, Elena Pinelli

**Affiliations:** ^1^Center for Infectious Disease Control, National Institute for Public Health and the Environment, Bilthoven, Netherlands; ^2^Department of Infectious Diseases and Immunology, Faculty of Veterinary Medicine, Utrecht University, Utrecht, Netherlands

**Keywords:** Reactive Oxygen Species (ROS), neutrophil, *Bordetella pertussis*, antibody, opsonization

## Abstract

Pertussis is a respiratory infection caused by the Gram-negative bacterium *Bordetella pertussis*. Despite high vaccination coverage this disease remains a public health concern worldwide. A better understanding of the protective immune responses to *B. pertussis* is required for the development of improved vaccines. The aim of this study was to determine the production of reactive oxygen species (ROS) by human neutrophils in response to *B. pertussis* and to determine the contribution of opsonizing antibodies from convalescent pertussis patients in this response. The serum samples from convalescent patients were taken at <3, 9, 18 and 36 months after diagnosis of pertussis. Also included were sera from healthy age-matched controls. We show that neutrophils produced high levels of ROS in response to opsonized, compared to non-opsonized, *B. pertussis* and that this effect was independent of the time the convalescent serum samples were taken. This indicates the presence of functional opsonizing antibodies up to 3 years after *B. pertussis* infection. While opsonization of *B. pertussis* with serum samples from uninfected controls also induced ROS production, sera from infected individuals induced significantly higher ROS levels. Spearman correlations analysis showed that IgG antibodies targeting fimbriae3 followed by pertactin, and BrkA correlate with ROS production. Additionally, we observed that neutrophils killed opsonized *B. pertussis* in a ROS-dependent manner. Searching for other antigen-specific antibodies from convalescent pertussis patients involved in ROS production by neutrophils may assist in the identification of novel antigens to improve the current pertussis vaccines.

## Introduction

Pertussis, commonly known as whooping cough, is a highly contagious respiratory disease that is transmitted *via* aerosolized respiratory droplets (Melvin et al., 2014; Nieves and Heininger, 2016). The causative agent of this disease is the Gram-negative bacterium *Bordetella pertussis*, which exclusively infects humans. Individuals from different age groups can develop pertussis resulting, among others, in severe coughing and in children it can even be fatal (Paddock et al., 2008; Yeung et al., 2017). Despite the availability of pertussis vaccines for over 60 years and high vaccination coverage, pertussis has been reemerging worldwide in the past three decades (Locht, 2016).

The innate immune system is the first line of defense against invading pathogens. Innate immune cells detect the presence of the pathogen and play an essential role in clearing an infection (Akira et al., 2006). Neutrophils are essential for protection against different respiratory bacterial pathogens such as, *Klebsiella pneumoniae* (Ye et al., 2001)*, Pseudomonas aeruginosa* (Tsai et al., 2000) *and Legionella pneumophila* (Tateda et al., 2001). These cells have various bactericidal effector mechanisms including, phagocytosis, degranulation (Dale et al., 2008), the release of neutrophil extracellular traps (Papayannopoulos, 2018) and the production of reactive oxygen species (ROS) (Nguyen et al., 2017). ROS is produced by the NADPH oxidase complex, a membrane bound enzyme complex located at the plasma or phagosomal membranes of neutrophils (Dupre-Crochet et al., 2013). Activation of the NADPH complex results in the release of ROS into the extracellular space or into the phagolysosome where it kills pathogens by, among others, directly damaging their cell membrane, proteins and DNA (Dupre-Crochet et al., 2013; Nathan and Cunningham-Bussel, 2013). Neutrophils have been shown to play a critical role in clearing a *B. pertussis* infection (Andreasen and Carbonetti, 2009; Eby et al., 2015). Despite the recruitment of neutrophils to the lungs of naive *B. pertussis* infected mice, neutrophils are unable to control bacterial growth early after infection. Only during the later stages of infection or after serum transfer from convalescent to naïve mice are neutrophils able to assist in clearing a *B. pertussis* infection (Andreasen and Carbonetti, 2009). This indicates that for bacterial killing by neutrophils, *B. pertussis* specific antibodies are required. *In vitro* studies have shown that neutrophils can phagocytose *B. pertussis* and efficiently kill these internalized bacteria (Lenz et al., 2000; Rodriguez et al., 2001). This bacterial internalization was enhanced upon neutrophil binding to IgG-opsonized *B. pertussis via* FcγRIIa and FcγRIIIb or to IgA-opsonized *B. pertussis via* the FcαR (Rodriguez et al., 2001).

The aim of the current study is to evaluate whether *B. pertussis* opsonization with serum from convalescent pertussis patients contribute to ROS production by human neutrophils and if so, for how long these antibodies remain present after diagnosis of the disease. Additionally, we set out to determine whether the ROS production contribute to killing of *B. pertussis* by neutrophils. To this end we made use of serum samples from convalescent pertussis patients (BP-serum) collected at multiple timepoints, from within 3 months to up to 3 years after initial diagnosis. In addition, we used serum samples from healthy age-matched control individuals (Ctrl-serum) who reported not to be diagnosed for any infectious disease in the past 12 months. IgG and IgA levels in corresponding plasma of all participants was measured against nine different *B. pertussis* antigens. We performed correlation analysis between these antibody levels and ROS production as an approach to identify which specific antibodies may contribute the most to the neutrophil ROS production. Findings indicate that *B. pertussis* opsonized with serum from pertussis patients 3 years after initial diagnosis, still elicited increased ROS production by neutrophils compared to the non-infected controls. In addition, we show that IgG antibodies to fimbriae 3 (Fim3) followed by pertactin (Prn) and Bordetella resistant to killing antigen (BrkA) positively correlate with ROS produced by neutrophils upon exposure to the opsonized bacteria and that the ROS production was important for *B. pertussis* killing by human neutrophils.

## Materials and Methods

### Ethical Statement

Participants were symptomatic, laboratory-confirmed pertussis (ex-)patients and healthy controls who declared to have no clinical history of infectious diseases for at least one year. These participants were part of a Dutch controlled longitudinal observational study started in 2015 (Immfact). This study was approved by the accredited review board METC UMC Utrecht (NL46795.094.13). All participants or parents/guardians of minor participants provided written informed consent. This study was conducted according to the principles described in the Declaration of Helsinki. For the collection of fresh human neutrophils and subsequent analyses, blood sample were obtained from a mini-donor system and all blood donors provided written informed consent. Blood samples were processed anonymously and the research goal, primary cell isolation, required no review by an accredited Medical Research Ethics Committee, as determined by the Dutch Central Committee on Research involving human subjects.

### Study Population and Serological Screening

**Figure 1:**
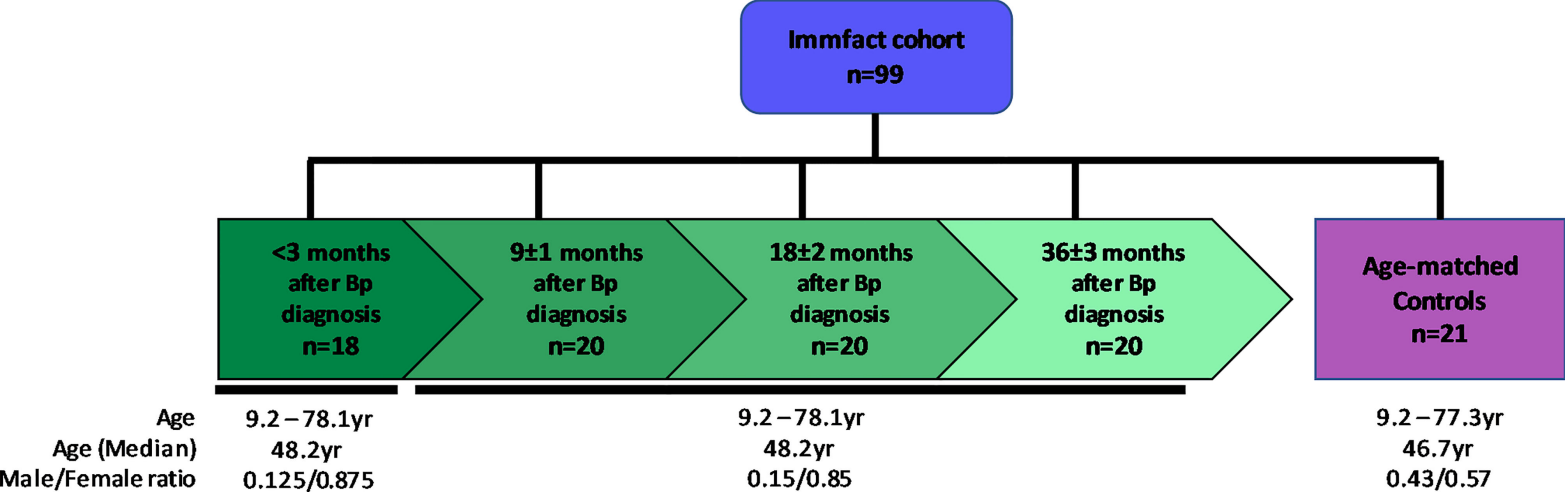
Schematic representation of the study population. Shown are the number (n) of serum samples in the convalescent pertussis ex-patients group (green) which were sampled in a longitudinal fashion at <3, 9, 18 and 36 months after diagnosis as well as that of the age matched control group (purple). Age median and range in years as well as male/female ratio are shown per sample group.

This study involves twenty pertussis (ex-)patients (age at inclusion 9.2 – 78.1 years, median 48.2 years, male/female ratio: 0.15/0.85) and 21 age-matched controls (age at inclusion 9.2 – 77.3 years, median 46.7 years, male/female ratio: 0.43/0.57) representing a wide spectrum of age groups. All participants were vaccinated according to the Dutch national immunization program, except for those born prior to implementation of the pertussis vaccination (1957) in the Netherlands. Coagulated and anti-coagulated peripheral blood was collected for serum and plasma isolation, respectively, and samples were stored at -20°C until use. The pertussis (ex-)patients were sampled in a longitudinal fashion at <3, 9 ± 1, 18 ± 2 and 36 ± 3 months after diagnosis of pertussis and controls were sampled once. For this study a total of 99 plasma samples were used which were tested for IgG and IgA antibody levels against nine *B. pertussis* antigenic specificities (Pieren et al, manuscript in preparation), using a previously described multiplex immune assay (van Twillert et al., 2017), extended to include in addition to pertussis toxin (Ptx), filamentous hemagglutinin (FHA), pertactin (Prn), outer membrane vesicles (OMV), and lipooligosaccharide (LOS), also fimbriae 2 (Fim2), fimbriae 3 (Fim3), virulence associated gene 8 (Vag8) and BrkA (**Supplementary Table 1**). For two pertussis (ex-)patients no samples were available at <3 months after diagnosis (**Figure 1**). For one pertussis (ex-)patient, serological data for IgA specific for Ptx, OMV and LOS at 18 months after diagnosis were missing as well as all IgA data from one healthy control participant. Healthy controls for this study were prescreened for the absence of a plasma IgG-Ptx serological response (< 20 IU/mL) (de Greeff et al., 2010; van Twillert et al., 2017).

### Bacterial Strains and Growth Conditions

In this study the naturally circulating B1917 *B. pertussis* strain, isolated from a Dutch pertussis patient in 2000, was used. To ensure consistency between experiments, flash freeze vials (FFVs) were prepared as previously described (Kroes et al., 2019). Before using the FFVs, they were spun down for 10 min at 16,000 x g and for the ROS assay resuspended in Hanks’ balanced salt solution (HBSS; Sigma-Aldrich) + 0.1% human serum albumin (HSA; Sanquin) and 50μM luminol (Sigma-Aldrich). For the killing assay the bacteria were resuspended in Iscove Modified Dulbecco Media (IMDM; Gibco) with 10% heat inactivated (HI) fetal calf serum (FCS; Hyclone).

### Neutrophil Isolation

Isolation of primary neutrophils was performed using fresh blood from multiple healthy donors. Neutrophils were isolated by using a Ficoll-Histopaque gradient method. In short, heparinized blood was diluted with an equal volume of PBS and layered onto a gradient of Ficoll-Paque (GE Healthcare Life Sciences) and Histopaque-1119 (Sigma-Aldrich). After centrifugation at 396g, for 20 minutes, neutrophils were collected from the Histopaque layer. After washing with HBSS containing 0.1% HSA, the neutrophils were subjected to hyperosmotic shock with ice-cold water for 30 seconds to lyse erythrocytes. Isolated neutrophils were washed again with HBSS + 0.1% HSA. Viability of neutrophils was verified after isolation by using the live-dead staining BD Horizon Fixable Viability Stain 780 (BD Biosciences) and was shown to be >95% for all experiments. Samples were acquired on the FACS Symphony A3 (BD Biosciences) flow cytometer and data analysis was done using FlowJo software (BD Biosciences).

### Bacterial Opsonization

The serum samples used for opsonization of *B. pertussis* were collected at <3, 9 ± 1, 18 ± 2 and 36 ± 3 months after pertussis diagnosis. Sera from un-infected age matched controls were also included. All serum samples were heat inactivated (HI) at 56°C for 30 min. For antibody-mediated opsonization, bacteria were incubated with 10% HI serum for 30 min at 37°C under gentle agitation. For normalization purposes, a HI reference serum sample from a positive pertussis patient was included. This reference serum was kindly provided by the Centre for Diagnostics and Laboratory Surveillance (IDS) at the National Institute for Public Health and the Environment.

### ROS Assay

Neutrophils were suspended in HBSS + 0.1% HSA in the presence of 50 μM luminol. A total of 200,000 neutrophils were seeded into a white 96-well microplate (Sigma-Aldrich). Cells were incubated with non-opsonized *B. pertussis*; HI Ctrl- or BP-serum only; or opsonized *B. pertussis* at multiplicity of infection (MOI) 10 in HBSS + 0.1% HSA. To inhibit ROS production, neutrophils were incubated with 5 µM VAS3947 NADPH oxidase (NOX) inhibitor VIII (Millipore) for 30 min prior to incubation with live *B. pertussis*. The concentration of the ROS inhibitor used was selected from previous titration experiments showing optimal ROS inhibition and neutrophil viability (data not shown). As a positive control for ROS production by neutrophils, cells were incubated with 5 ng/ml PMA (**Supplementary Figure 1**). For normalizing purposes, a reference serum was included in every plate when measuring ROS production to correct for experiment, plate and neutrophil donor variability. The production of ROS was determined in real-time by measuring chemiluminescence with a TriStar² LB 942 multimode microplate reader (Berthold Technologies). Light emission was recorded in relative light units (RLU’s) for 30 minutes at 37°C. For representation purposes area under the curve (AUC) values were determined using the area under curve function in GraphPad prism 9.1.0 using standard parameters.

### Killing Assay

Opsonized or non-opsonized *B. pertussis* at MOI 10 were incubated in the presence or absence of 200,000 neutrophils in IMDM with 10% HI FCS (Hyclone) for 4 hours at 37°C. When indicated, neutrophils were incubated with 5 nM VAS2870 NOX inhibitor III (Millipore) for 30 min prior to incubation with live *B. pertussis* to inhibit ROS production. After 4 hour incubation the samples were treated with sterile water at pH 11 for 5 minutes to lyse neutrophils and release surviving bacteria (Decleva et al., 2006). Lysates were further diluted and each dilution was plated on Bordet Gengou (BG) agar plates containing 15% sheep blood (BD Bioscience) and incubated at 35°C and 5% CO_2_ for 5 days after which bacterial colonies were counted using a ProtoCOL 3 colony counting system (Symbiosis). Only bacterial counts between 15 and 305 colonies were included for further analysis. To correct for the different neutrophil donors used in the various experiments, the data shown (% CFU) is relative to the bacterial counts when using neutrophils incubated with non-opsonized *B. pertussis*.

### Statistical Analysis

Statistical analysis was performed as previously described (Kroes et al., 2021). Briefly, the permutation based exact Wilcoxon-Mann-Whitney test was used (*wilcox_test* function from *rstatix* R package). p-values were corrected for multiple testing by the Benjamini-Hochberg method.

Effect sizes (ES), indicating how big the differences between two groups are, were calculated as Cliff’s delta. The established thresholds are: small, <0.28; medium, <0.43; large, <0.7 and very large >= 7 (Vargha and Delaney, 2000). Comparisons with both padj <0.05 and ES either medium or higher, as well as padj <0.1 and ES either large or very large are considered significantly different.

When measuring ROS production using the control sera, one serum sample induced 8 times more ROS than the average of the group. The presence of outliers was explored for this group by Grubbs’ test (GraphPad Prism (version 9.1.0)), which qualified that unique sample as outlier, and was consequently eliminated from the analysis.

For correlation analysis the *rcorr* function from the *Hmisc* R package was used to calculate the spearman correlations between ROS production and antibody levels. Spearman’s Rho rank (r_s_) and approximate p-values were provided. The *corrplot* function from *corrplot* R package was used to represent the above mentioned correlations. p-values were corrected for multiple testing by the Benjamini-Hochberg method. A correlation was considered significant at an adjusted p-value <0.05. If not significant, the corresponding cell in the plot appeared empty.

## Results

### Long-Lasting Antibodies Induce Neutrophil ROS Production

**Figure 2:**
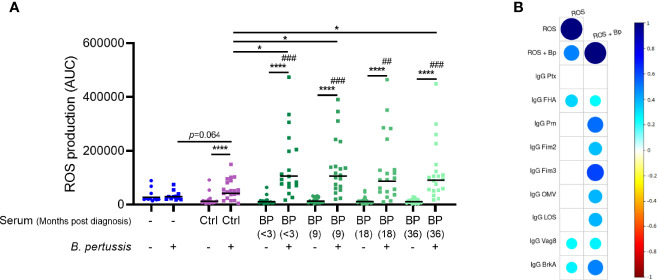
Neutrophil ROS production induced by opsonized *B. pertussis*. **(A)** ROS production was measured in real-time for 30 minutes after incubation of neutrophils in the presence (squares) or absence (circles) of (opsonized) *B. pertussis* at MOI 10. Data is represented as individual AUC values with geometric mean. # indicate significance to “No serum, No *B. pertussis*” condition (blue circle). Ctrl (purple) indicates serum derived from healthy control individuals (n=21) and BP (green) indicate serum derived from convalescent pertussis patients (n=18 for timepoint <3 months, n=20 for remaining timepoints). *p <0.05, ^##^p < 0.01, ^###^p < 0.001, ****p < 0.0001. **(B)** Spearman correlations plots were performed between neutrophil ROS production at all time points and the levels of nine different antigen-specific IgG antibody levels in plasma of the same convalescent pertussis patients. The ROS production was measured after incubating neutrophils with Bp-serum only (ROS) or with *B. pertussis* opsonized with BP-serum (ROS + Bp). Circles indicate a correlation with adjusted p < 0.05 and circle colors and size indicate the correlation r_s_ value.

To investigate ROS production by neutrophils in response to *B. pertussis* we isolated neutrophils from the blood of healthy individuals and incubated these with live *B. pertussis* in the presence of luminol, allowing the measurement of ROS production. In the absence of antibodies, we found no evidence that neutrophils produce additional ROS upon encountering *B. pertussis* (**Figure 2A**). Previous studies in mice highlighted a critical role for *B. pertussis*-specific antibodies in the control of *B. pertussis* infection by neutrophils (Andreasen and Carbonetti, 2009). Therefore, we set out to elucidate the role of *B. pertussis*-specific antibodies in the production of ROS by human neutrophils. To this end, we opsonized live *B. pertussis* with HI serum from laboratory-confirmed pertussis patients longitudinally collected <3 and 9, 18 and 36 months after the diagnosis of symptomatic pertussis (BP-serum) or HI serum of healthy age-matched control individuals (Ctrl-serum; **Figure 1**). *Bordetella pertussis* opsonized with BP-serum collected at any timepoint after diagnosis significantly induced ROS production by human neutrophils (**Figure 2A**) which did not wane up to 3 years after the acute stage of the disease. While *B. pertussis* opsonized with Ctrl-serum also induced ROS production, this ROS production was significantly lower compared to *B. pertussis* opsonized with BP-serum (**Figure 2A**). Incubation of neutrophils with serum alone did not induce any ROS production above background levels (**Figure 2A**). To have an indication of which antibodies are most responsible for the increased ROS production against the BP-serum opsonized bacteria, we made use of a dataset of IgG and IgA antibody levels targeting Ptx, FHA, Prn, Fim2, Fim3, Bp-OMV, LOS, Vag8 and BrkA in corresponding plasma samples (**Supplementary Table 1**). Correlation analysis was performed between the individual antigen-specific antibody levels and the neutrophil ROS production induced by BP-serum only or by *B. pertussis* opsonized with the BP-serum. Findings indicate a positive correlation between ROS production by neutrophils and IgG antibodies targeting Fim3 followed by Prn and BrkA (**Figure 2B**). These correlations were strong and highly significant (r_s_ >0.5, p-value <1.05E-06). Weaker correlations were found for all other antigen-specific antibody levels, except those targeting Ptx for which no correlation at all was found. For the antigen-specific IgA antibodies, no positive correlations were found with the neutrophil ROS production (data not shown).

### ROS Production Is Important in Bacterial Killing by Neutrophils

**Figure 3:**
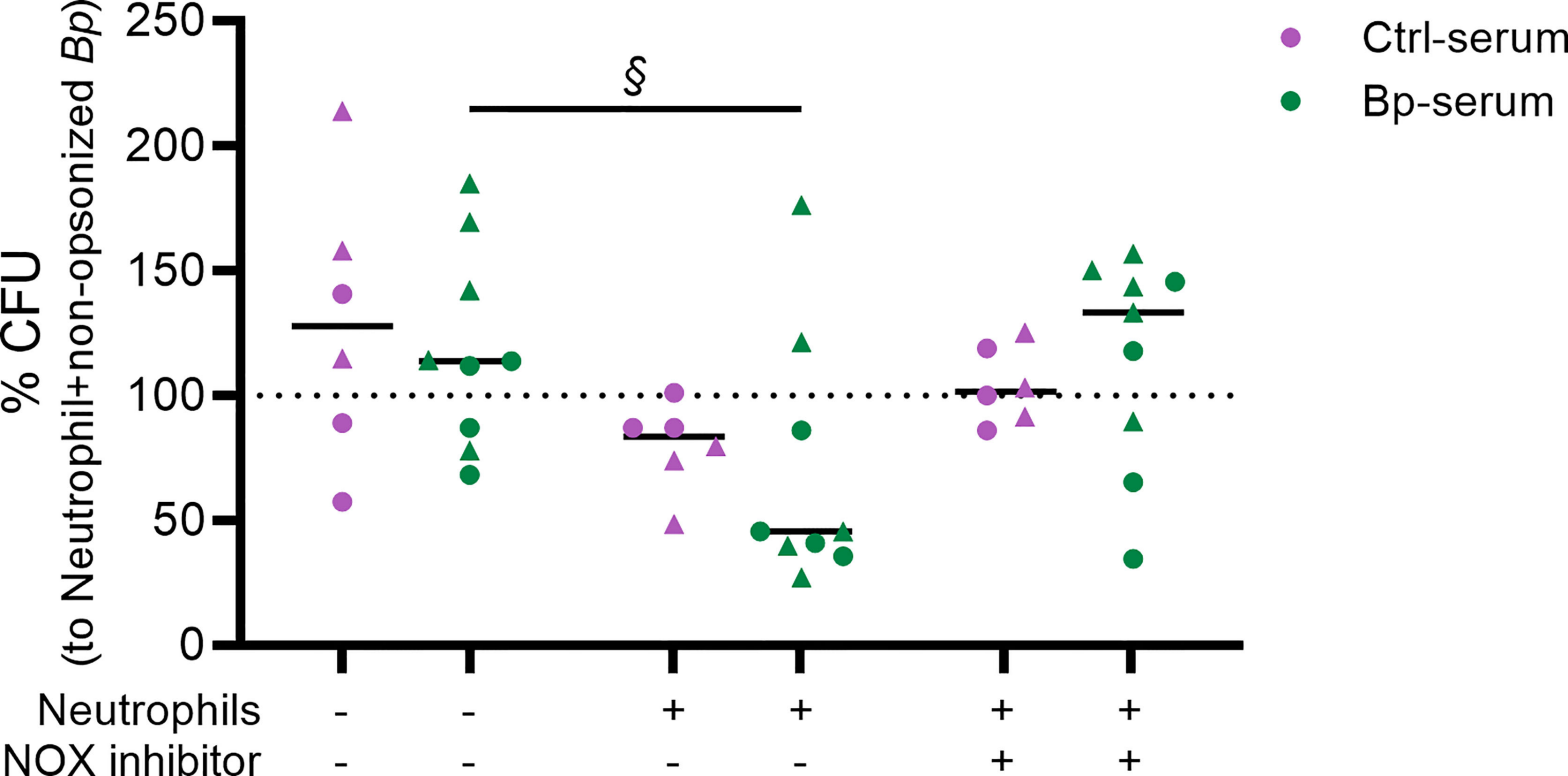
Neutrophil killing of opsonized *B. pertussis* and the role of ROS in this process. Live *B. pertussis* (MOI 10) were opsonized with serum samples from healthy controls (Ctrl-serum) shown in purple or from convalescent pertussis ex-patients (BP-serum) (<3 month after diagnosis) in green. The opsonized bacteria were incubated in the presence or absence of neutrophils and/or NOX inhibitor for 4 hours after which neutrophils were lysed and total CFU were determined by plating on BG plates. Bacterial growth is represented as % CFU which was calculated relative to CFU from non-opsonized bacteria incubated with neutrophils (dotted line). § p = 0.075 + ES = 0.605 indicating significant differences. The data represented are of 2 independent experiments (each represented by symbol shapes).

We then investigated whether primary human neutrophils were able to kill *B. pertussis* and the role for ROS in this process. **Figure 3** shows the % CFU for live *B. pertussis* opsonized with either BP or Ctrl sera in the presence or absence of neutrophils. The % CFU was calculated relative to the CFU values obtained when using non-opsonized bacteria incubated with neutrophils from the corresponding donor (dotted line). Results indicate that in the presence of neutrophils a significant enhanced killing of *B. pertussis* (less % CFU) was observed when the bacteria were opsonized with BP-sera (green) (p = 0.075, large effect size (ES) = 0.605) compared to the opsonized *B. pertussis* in the absence of neutrophils. No significant difference was observed when the bacteria were opsonized with Ctrl-sera (purple) and incubated with neutrophils compared to the opsonized bacteria only

To determine the contribution of ROS production in bacterial killing, we incubated the neutrophils with the VAS287 NOX inhibitor III which blocks both intra- and extracellular ROS production. Validation of the inhibitory effect of the VAS287 NOX inhibitor III on ROS production by human neutrophils was performed (**Supplementary Figure 1**). **Figure 3** shows a reduction of bacterial killing in the presence of the NOX inhibitor, as indicated by an increased trend in % CFU which value is comparable to that obtained when the opsonized bacteria were cultured in the absence of neutrophils. These findings suggest an important role for neutrophil derived ROS in killing of *B. pertussis* opsonized with sera from convalescent pertussis patients.

## Discussion

In the present study we show that human neutrophils readily produce ROS upon incubation with live *B. pertussis* opsonized by human serum obtained from convalescent pertussis patients up to 3 years after diagnosis of the disease. It is worth mentioning that after an initial rise of specific antibody levels in pertussis patients, these decline within the first 3 months following diagnosis (van Twillert et al., 2017). Therefore, it may be that higher levels of ROS are produced by neutrophils during the acute stage of pertussis. In addition, we showed that opsonization of *B. pertussis* using the sera from healthy controls also increased neutrophil ROS production, although this was significantly lower than the ROS induced by convalescent pertussis patient serum. That the healthy control serum can also induce ROS production by neutrophils may be due to the fact that these individuals have been vaccinated against pertussis during their childhood. This suggests that not only antibodies induced by infection, but also those by vaccination can opsonize *B. pertussis* and enhance neutrophil ROS production. Also to bear in mind are the asymptomatic infections. Due to the widespread circulation of *B. pertussis*, also unvaccinated individuals have been reported to have specific antibodies against this bacterium (de Greeff et al., 2010). Although the findings described here suggest that antibody opsonization is important for increased production of ROS by neutrophils, it is important to confirm these results by blocking Fc receptors as well as using purified IgG and IgA fractions.

Next to an increase in ROS production, we found that opsonization of *B. pertussis* with sera of convalescent pertussis patients resulted in higher bacterial killing compared to bacteria opsonized with control sera or non-opsonized bacteria. Furthermore, ROS appears to play a role in this process as its inhibition interferes with bacterial killing. This corroborates previous studies which showed that ROS production was essential in the TLR4-dependent innate clearance of *B. pertussis* in a murine infection model (Zurita et al., 2013). Future studies should include the use of a higher number of serum samples from convalescent patients as well as investigating the exact mechanism(s) by which ROS production promotes bacterial killing by human neutrophils. Whether neutrophils secrete inflammatory cytokines during this process that enhances bacterial killing as well as the role for other innate cells, such as alveolar macrophages, are some of the questions that remain to be addressed. Understanding the mechanisms involved in protective immunity is essential for the improvement of vaccines that prevent nasal colonization and *B. pertussis* transmission.

By correlating the plasma IgG and IgA antibody levels targeting Ptx, FHA, Prn, Fim2, Fim3, Bp-OMV, LOS, Vag8 and BrkA with ROS production we have been able to identify anti-Fim3, anti-Prn and anti-BrkA IgG as putative relevant antibodies inducing neutrophil ROS production. This suggests that antibodies targeting these antigens are important for the bactericidal activity of human neutrophils. Previous studies indicate that vaccination with an acellular pertussis (aP) vaccine induced bactericidal antibodies that are specific for Prn (Lesne et al., 2020). This was suggested as a driving force in the emergence of Prn-deficient *B. pertussis* strains in countries using aP vaccination. Hence the development of a vaccine capable of inducing bactericidal antibodies against multiple antigens to prevent vaccine-induced bacterial adaptation and vaccine evasion is imperative. By screening convalescent sera for antibodies targeting as many as possible surface exposed and secreted *B. pertussis* proteins (Raeven et al., 2020) and correlating them with neutrophil ROS production may assist in the identification of novel antigens that are important in neutrophil ROS production and bacterial killing. This approach could also help to supplement current aP vaccines to induce a stronger protective antibody response targeting multiple antigens. However, since excessive release of ROS can damage host tissue this response has to be closely monitored (Laforge et al., 2020). To-date, there is no reliable correlate of protection (CoP) described for pertussis. It is widely accepted that merely analyzing antibody levels will not reveal a CoP and that evaluating the functionality of antibodies is an important next step in the search for a CoP (Hovingh et al., 2018). Measuring ROS production by neutrophils in response to *B. pertussis* opsonized with sera of vaccinated individuals should be further explored as a correlate of protection.

## Data Availability Statement

The original contributions presented in the study are included in the article/**Supplementary Material**. Further inquiries can be directed to the corresponding author.

## Ethics Statement

The studies involving human participants were reviewed and approved by METC UMC Utrecht. Written informed consent to participate in this study was provided by the participants’ legal guardian/next of kin.

## Author Contributions

EP and MK contributed to conception and design of the study. CvE, BK, and DP organized the database. AM-B performed the statistical analysis. MK, RJ, and LvV performed experiments. EP, MK, and LvV wrote the first draft of the manuscript. All authors contributed to manuscript revision, read, and approved the submitted version.

## Funding

This work was supported by the Dutch Ministry of Health, Welfare and Sport.

## Conflict of Interest

The authors declare that the research was conducted in the absence of any commercial or financial relationships that could be construed as a potential conflict of interest.

## Publisher’s Note

All claims expressed in this article are solely those of the authors and do not necessarily represent those of their affiliated organizations, or those of the publisher, the editors and the reviewers. Any product that may be evaluated in this article, or claim that may be made by its manufacturer, is not guaranteed or endorsed by the publisher.

## Supplementary Material

Supplementary Table 1 Plasma IgG and IgA antibody levels targeting Ptx, FHA, Prn, Fim2, Fim3, Bp-OMV, LOS, Vag8 and BrkA.

Supplementary Figure 1 Inhibition of ROS production by the NOX inhibitor. ROS production was measured in real-time for 30 minutes after incubation of neutrophils in the presence or absence of 5 ng/ml PMA, *B. pertussis* (MOI 10), *B. pertussis* opsonized with a reference serum from a pertussis patient and/or the VAS3947 NOX inhibitor VIII. Data are represented as mean ± SEM.
