# Supplementary figures and images for: Long Lasting Antibodies From Convalescent Pertussis Patients Induce ROS Production and Bacterial Killing by Human Neutrophils
Source: Front Cell Infect Microbiol. 2022 May 12;12:888412. doi: 10.3389/fcimb.2022.888412 (PMC9135168; doi:10.3389/fcimb.2022.888412)

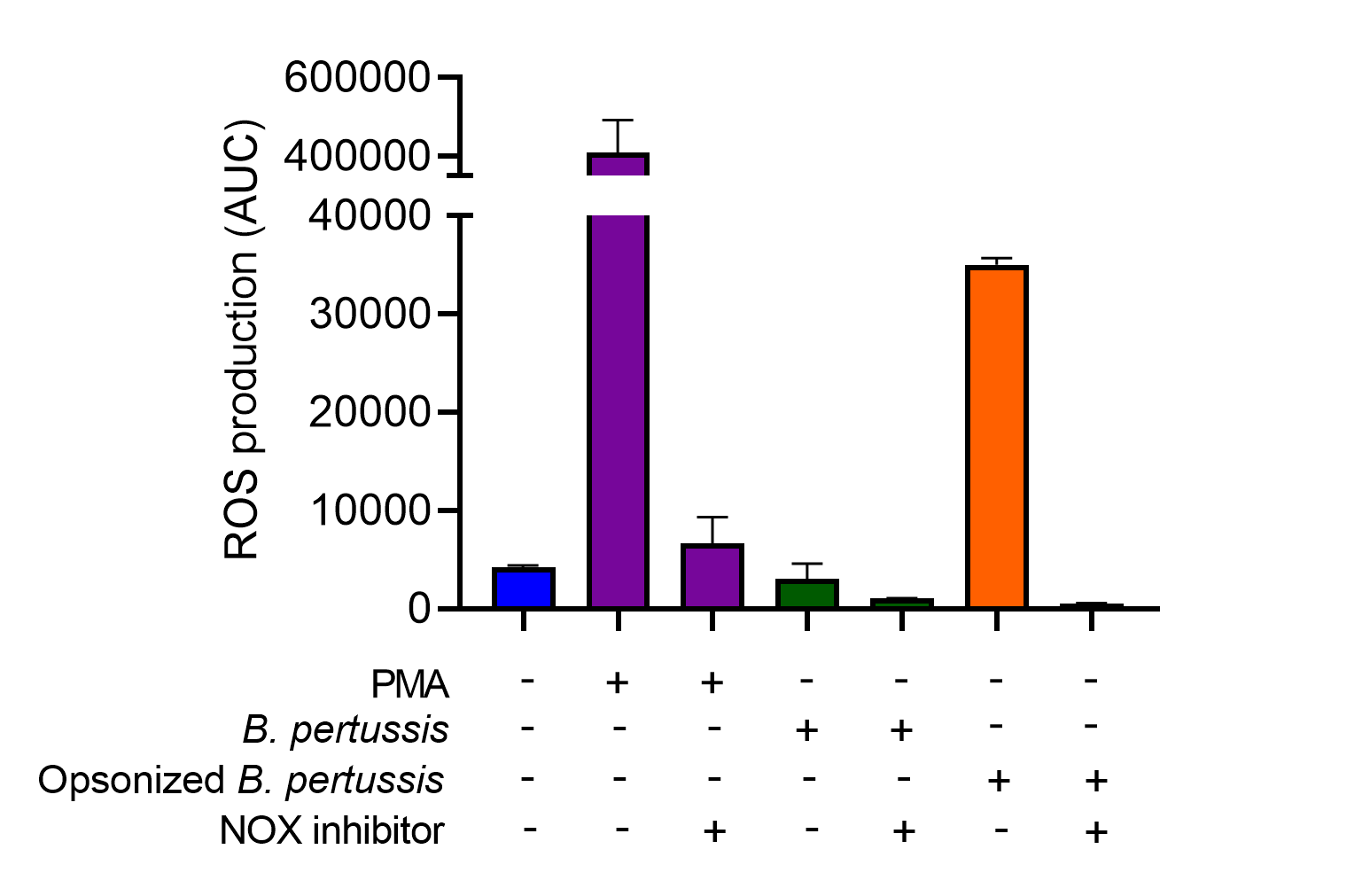

Supplement: Supplementary Figure 1 — Inhibition of ROS production by the NOX inhibitor. ROS production was measured in real-time for 30 minutes after incubation of neutrophils in the presence or absence of 5 ng/ml PMA, B. pertussis (MOI 10), B. pertussis opsonized with a reference serum from a pertussis patient and/or the VAS3947 NOX inhibitor VIII. Data are represented as mean ± SEM. [file Image_1.tif]
